# Microstructure, Texture, and Mechanical Properties of Friction Stir Spot-Welded AA5052-H32: Influence of Tool Rotation Rate

**DOI:** 10.3390/ma16093423

**Published:** 2023-04-27

**Authors:** Mohamed M. Z. Ahmed, Mohamed M. El-Sayed Seleman, Ibrahim Albaijan, Ali Abd El-Aty

**Affiliations:** 1 Department of Mechanical Engineering, College of Engineering at Al Kharj, Prince Sattam Bin Abdulaziz University, Al Kharj 11942, Saudi Arabia; i.albaijan@psau.edu.sa (I.A.); a.hassibelnaby@psau.edu.sa (A.A.E.-A.); 2 Department of Metallurgical and Materials Engineering, Faculty of Petroleum and Mining Engineering, Suez University, Suez 43512, Egypt; mohamed.elnagar@suezuniv.edu.eg; 3 Mechanical Engineering Department, Faculty of Engineering-Helwan, Helwan University, Cairo 11795, Egypt

**Keywords:** AA5052-H32 aluminum alloy, friction stir spot welding, rotation speed, hardness, joint strength, grain structure, crystallographic texture, electron back scattered diffraction (EBSD)

## Abstract

Friction stir spot welding (FSSW) of similar AA5052-H32 joints has numerous benefits in shipbuilding, aerospace, and automotive structural applications. In addition, studying the role of tool rotation speed on the microstructure features, achieved textures, and joint performance of the friction stir spot-welded (FSSWed) joint still needs more systematic research. Different FSSWed AA5052-H32 lap joints of 4 mm thickness were produced at different heat inputs using three tool rotation speeds of 1500, 1000, and 500 rpm at a constant dwell time of 2 s. The applied thermal heat inputs for achieving the FSSW processes were calculated. The produced joints were characterized by their appearance, macrostructures, microstructures, and mechanical properties (hardness contour maps and maximum tensile–shear load) at room temperature. The grain structure and texture developed for all the FSSWed joints were deeply investigated using an advanced electron backscattering diffraction (EBSD) technique and compared with the base material (BM). The main results showed that the average hardness value of the stir zone (SZ) in the welded joints is higher than that in the AA5052-H32 BM for all applied rotation speeds, and it decreases as the rotation speed increases from 500 to 1000 rpm. This SZ enhancement in hardness compared to the BM cold-rolled grain structure is caused by the high grain refining due to the dynamic recrystallization associated with the FSSW. The average grain size values of the stir zones are 11, 9, and 4 µm for the FSSWed joints processed at 1500, 1000, and 500 rpm, respectively, while the BM average grain size is 40 µm. The simple shear texture with B/-B components mainly dominates the texture. Compared to the welded joints, the joint processed at 500 rpm and a 2 s duration time attains the highest tensile-shear load value of 4330 N. This value decreases with increasing rotation speed to reach 2569 N at a rotation speed of 1500. After tensile testing of the FSSWed joints, the fracture surface was also examined and discussed.

## 1. Introduction

Using lightweight materials in transportation industries has become a popular strategy for reducing power use and fuel consumption [1,2,3]. Aluminum-based materials can satisfy the requirements for developing lightweight vehicles since aluminum has lower specific gravity than steel, high corrosion resistance, a high strength-to-weight ratio, good weldability, impact resistance, and recycling potential. The Al-Mg series alloys (5XXX Al alloys) have drawn significant attention for automotive and marine structural applications [4,5,6]. One of the most important alloys in this group is AA5052 aluminum alloy. This alloy is widely utilized in the manufacture of automotive components, vessels, and ships, and it may be welded using different fusion welding methods, including metal inert gas (MIG) [7,8], tungsten inert gas (TIG) [9,10], and resistance spot welding (RSW) [11,12]. These conventional welding processes depend on phase regulator transistorized power sources, which consume a lot of energy and require extended cooling times. Furthermore, welding defects such as micro-voids, solidification cracking, and joint distortion cannot be prevented [13,14,15]. To overcome these fusion welding defects, friction-based, solid-state welding technologies such as friction stir welding (FSW) [16,17] and its derivative friction stir spot welding (FSSW) [18,19,20] can be utilized.

The FSSW process is a new development in FSW, which was suggested and applied by the Mazda motor corporation in 1993 [21]. FSSW is a solid-state joining technology that produces a spot weld by plunging the rotating tool into and out of the overlapping sheet materials at a single location. It has attracted the attention of many researchers due to its advantages, such as high joint efficiency, low joint distortion, low cost, ease of handling, and a clean working environment [22].

The FSSW procedure variables, including tool type and profile [23,24], rotation speed [25,26,27], plunge rate [28,29,30], and dwell time [31,32,33], govern the joint efficiency of friction stir spot welds. FSSW has been successfully utilized to weld various kinds of aluminum alloys, including AA7050 [34], AA6082-T6 [35], AA6061-T6 [36], AA6061-T4 [37], AA5182-O [38], AA5052-H112 [27], AA5052-H32 [30], AA3003-H12 [39], and AA2024-T3 [40]. The tool geometry results in the typical welded structure of the FSSW and determines the pattern of material flow during the stirring process. Furthermore, the tool geometry influences the hook dimensions and stir zone width [41,42,43,44]. During the welding process at a constant rotation speed, the heat generation, material plasticization around the pin, weld shape, and, consequently, the welded joint performance are governed by the dwell time and tool penetration [28,45]. When the dwell time is kept constant, the tool rotation speed is considered one of the crucial FSSW parameters [46,47].

The impact of tool rotation speed on the joint quality of the FSSW materials has been studied in some works [38,48,49,50]. These studies have found that rotation speed affects welding performance in various ways. Zhang et al. [30] analyzed tensile test results from FSSW AA5052-H112 lap joints and concluded that the joint performance decreases with increasing the rotation speed. Cao and Jahazi [51] concluded that the joint strength in the FSSW AZ31B-H24 magnesium alloy welds initially increases with increasing tool rotation speed but decreases with a further increase. Rojikin et al. [52] also reported a similar trend for the FSSW dissimilar AA5052-steel SS400 joints. Ahmed et al. [53] remarked a noticeable heat influence on the joint surface quality of the FSSW high-manganese TWIP steel (0.6 wt.% C, 22 wt.% Mn, 0.3 wt.% V) lap joints around the welded region that expanded into a sizable area with an increase in rotation speed.

Based on the above literature, the FSSW procedure was accomplished with severe plastic deformation, and the quality of the formed welds was considerably impacted by the quantity of heat input used. Furthermore, because FSSW is achieved in a shorter period of time than FSW, it promotes less heat-input energy. Thus, it is advised to use a high heat input to enhance the joint quality of friction stir welds. Moreover, aluminum alloys possess excellent thermal conductivity, and as a result, the high heat input may result in a coarse microstructure in the stir zone and then a reduced joint strength of the spot welds. Additionally, optimizing the tool rotation speed is a challenge to provide an optimum heat input and well-structured joints, especially at a short dwell time.

Thus, understanding the effect of tool rotation speed on microstructure features and its impact on the mechanical properties of AA5052-H32 FSSW joints still requires a thorough investigation, particularly for a wide range of tool rotation speeds and a short duration time. Consequently, the objectives of the current work are to study the applicability of utilizing FSSW to join AA5052-H32 alloy sheets in 4 mm similar lap joints at the short dwell time of 2 s and various tool rotation rates of 500, 1000, and 1500 rpm. In addition, this work aimed to evaluate the produced joint performance for the different introduced heat inputs by the analysis of the achieved microstructural features in terms of grain size, misorientation angles, and textures. To achieve these objectives, 2 mm thick AA 5052 H32 cold-rolled aluminum alloy strips were FSSWed at different rotation speeds (500, 1000, and 1500 rpm) while applying a constant dwell time of 2 s to produce 4 mm thick similar spot lap joints. The applied thermal heat inputs for achieving the FSSW joints were determined. The joint appearance was evaluated. Finally, the mechanical properties of the joined specimens in terms of hardness map and maximum load-carrying capacity were examined and interpreted in light of an extensive EBSD study on the microstructure features and the crystallographic texture.

## 2. Experiments and Procedures

### 2.1. Base Material

The starting materials utilized in the current study were AA5052-H32 alloy sheets with a thickness of 2 mm, a length of 1000 mm, and a width of 1000 mm. The sheets were produced by Misr Aluminum Company “Egyptalum”, Egypt. The chemical composition and mechanical properties of the AA 5052-H32 Al alloy base material (BM) are given in Table 1.

### 2.2. FSSW Experiments

**Figure 1:**
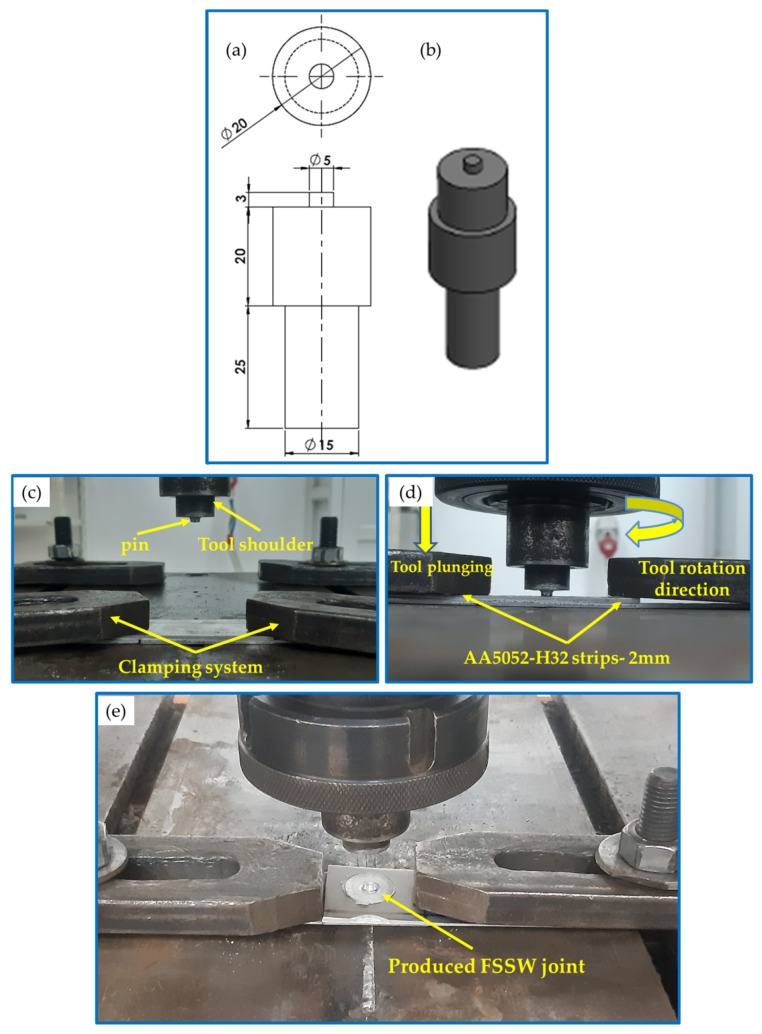
(**a**) Schematic drawing, (**b**) isometric image showing the FSSW tool (all dimensions are in mm), and (**c**–**e**) FSSW stages.

The AA 5052-H32 FSSW joints were processed utilizing a full-automatic FSW/FSSW machine (EG-FSW-M1) [54]. For the FSSW experiments, the sheets of AA 5052-H32 Al alloy were cut into strips with dimensions of 100 × 30 × 2 mm (length × width × thickness). These 5052 Al strips were jointed at various tool rotation speeds of 500, 1000, and 1500 rpm and a 2 s holding time in similar lap joints with a 30 mm overlap area. Other FSSW process variables, including tilt angle, plunge depth, and plunge rate, were kept constant at 0°, 3.2 mm, and 0.1 mm/s, respectively. The tool used in this study was made of hot-worked steel (AISI H13) and designed to have a cylindrical pin and a flat shoulder. The tool dimensions in terms of shoulder diameter, pin length, and pin diameter were 20, 3, and 5 mm, respectively, as given in Figure 1. Based on the previous works [43,55], the tool material and design were chosen. Aydin et al. [55] compared the role of various pin shapes (cylindrical, cylindrical with grooves, triangular, conical, and hexagonal) on the joint strength and the welded zone area for the FSSW AA6082-T6 joints at different dwell times and reported that the welds achieved using the cylindrical pins produced a wider effective weld width with a higher joint strength compared to the other pin profiles. In addition, Hirasawa et al. [43] concluded that the temperature distribution is axisymmetric during the stirring process when applying the cylindrical pin.

### 2.3. Spot-Welded Joints Characterization

**Figure 2:**
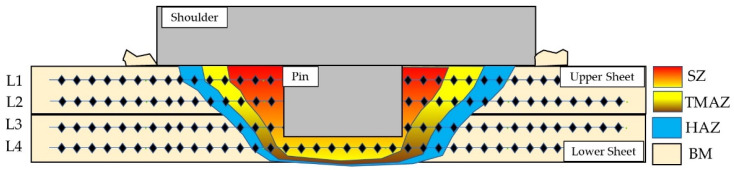
Schematic showing the target zones for hardness measurements during the hardness test performed on the FSSW AA5052 lap joints.

A macrostructure and microstructure examination and a hardness evaluation were performed on the cross-section of the FSSWed joints. SiC papers were used to grind all specimens to a 2400-grit level. This process was followed by polishing in the presence of distilled water (DW) using a polishing cloth and 0.05 µm alumina paste. Etching of the surface-polished specimens was achieved using Keller’s reagent (6 mL nitric acid (HNO_3_), 3 mL hydrofluoric (HF), and 95 mL DW). Microstructural investigations were accomplished using an Olympus optical microscope (model BX41M-LED, Olympus, Tokyo, Japan). Moreover, the FSSW joints and BM microstructures were deeply investigated using an electron back-scattered diffraction (EBSD) technique equipped with a Quanta FEG 250 SEM FEI Company, Hillsboro, OR, USA. For the EBSD examination, the BM and the welded specimens were mechanically polished, then electro-polished at a temperature of −15 °C and 14 V for 1 h. The electrolyte solution was 70 vol.% methanol (CH_3_OH) and 30 vol.% of HNO_3_. Post-processing of the EBSD data was carried out using OIM software version OIM7.3, and for the grain size calculation, any two points paired with misorientation exceeding 2 degrees were considered a boundary. To evaluate the hardness values of the BM and the different FSSW zones for the different welding conditions, hardness testing was performed on the cross-sections of the FSSW lap joints, and the results were used to plot hardness contour maps. Hardness was measured using a Vickers hardness testing machine (Type HWDV-75, TTS Unlimited, Osaka, Japan) at the testing conditions of 5 N load and 15 s holding time. The cross-sections of the FSSW AA 5052-H32 joint were divided into four lines to represent the two welded Al strips, as given in Figure 2. The spacing between each pair of indentations was adjusted to 0.75 mm.

**Figure 3:**
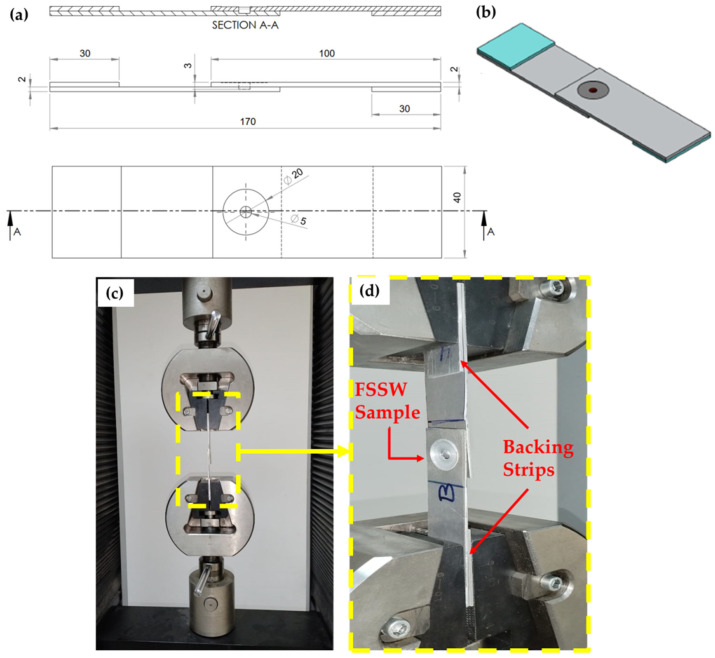
(**a**) Drawing and isometric showing the tensile–shear test specimen (**b**). Photographic image of the joint specimen during the tensile-shear test, and (**c**) High magnification of (**d**).

The tensile–shear tests were performed at room temperature using a universal tensile testing instrument (Model WDW-300D, Guangdong, China). For each tensile–shear test, at least three specimens were examined. All the tensile experiments were performed at 0.05 mm/sec crosshead speed. Figure 3 presents a drawing showing the tensile–shear test sample of the FSSWed AA5052 Al alloy joint. Two backing AA5052-H32 strips (30 mm length, 30 mm width, and 2 mm thickness) were used to ensure the applied axial load during tensile–shear testing. The fractured surfaces of the failed FSSWed joints were also examined using the SEM-Quanta FEG 250.

## 3. Results and Discussion

### 3.1. Joint Appearance

**Figure 4:**
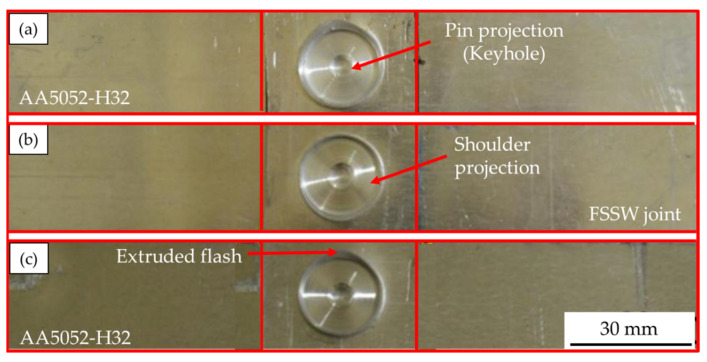
The top appearance of the AA5052-H32 FSSW joints produced at a 2 s dwell time and different rotation speeds of (**a**) 500, (**b**) 1000, and (**c**) 1500 rpm.

The top surfaces of the FSSW AA5052-H32 joints produced at a 2 s dwell time and rotation speeds of 500, 1000, and 1500 rpm are shown in Figure 4. It can be concluded that the chosen FSSW parameters are able to produce 4 mm thickness AA5052-H32 lap joints without any significant joint distortion. The formed keyhole and circular indentation of the shoulder projection, which serves as identifying characteristics of the FSSW joints, are detected for all the applied rotation speeds. During the FSSW process, the temperature rises as the rotational speed increases, which in turn lowers the viscosity of the material being stirred. This causes the shoulder projection depth to increase, the potential for a flash to form, and the thickness of the weld beneath the pin to decrease [56]. The depth of shoulder projection increases with the increase in the rotation speeds from 500 to 1500, as shown in Figure 4a–c, respectively.

### 3.2. Heat Input Energy and Cross-Section Macrographs of the Spot-Welded Joints

**Figure 5:**
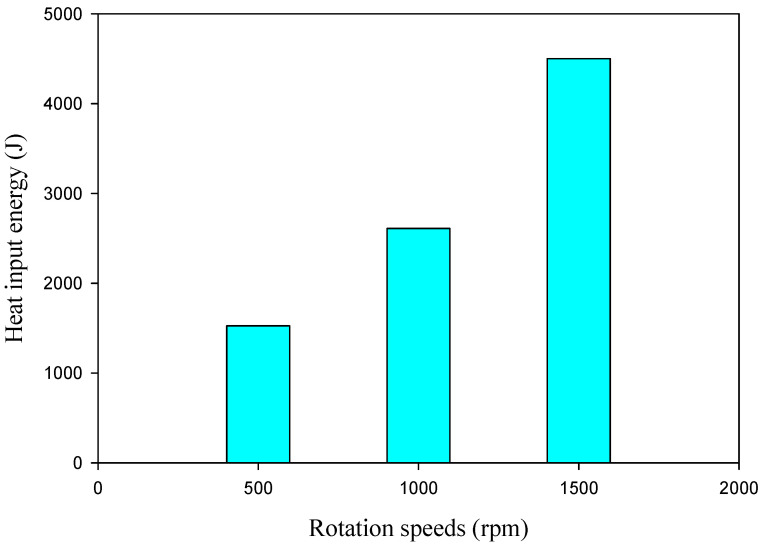
The heat input energy versus rotation speed to produce the AA5052-H32 FSSW joints at a 2 s dwell time.

A previous work [57] indicated that FSSW variables and their influence on introduced heat input in SZ significantly affect the joint quality and material flow. The generated heat input is the sum of the frictional heat input developed at the tool shoulder contact area and pin surrounding area, and it is directly governed by a number of parameters, including the rotation speed, tool geometry, dwell time, plunge depth, and the interface friction coefficient between the tool and the stirring material [56]. The heat input energies for producing the AA5052-H32 welded joints at a 2s dwell time and various tool rotation speeds of 500, 1000, and 1500 rpm were calculated using the Equations mentioned in the previous work [57], and the obtained results are plotted in Figure 5. During the FSSW, it can be shown that raising the rotational speed from 500 to 1500 rpm increases the heat input created from 1525 to 4500 J, respectively [58].

**Figure 6:**
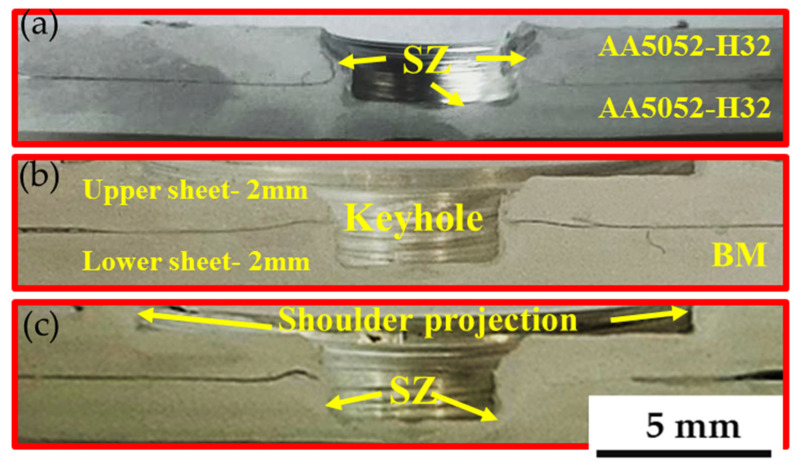
Typical macrographs showing the FSSW joint cross-sections processed at different rotation speeds of (**a**) 500, (**b**) 1000, and (**c**) 1500 rpm and a constant dwell time of 2 s.

Figure 6 depicts the macrographs of the cross-sectional FSSWed AA5052-H32 specimens. It is noted that the suggested welding variables yield defect-free joints. In addition, at the chosen dwell time of 2 s, the SZ increases with the increase in rotation speed from 500 to 1000 rpm. At the applied rotation speeds of (a) 500, (b) 1000, and (c) 1500 rpm, the interface between the overlapped AA5052-H32 aluminum strips is spot welded as a result of severe plastic deformation and material movement around the rotating pin at a constant dwell time of 2 s, as shown in Figure 6. Furthermore, a small flash is noticed at a joint processed at the highest heat input (Figure 5) with a remarked increase in the shoulder projection depth (Figure 6c) [59]. The resulting FSSWed joints have a distinctive keyhole in the center of the spot weld, which expresses pin indentation when the welding process is completed. This keyhole accurately depicts the pin geometry and dimensions and a slight increase with the increase in rotation speed due to increasing the material softening around the rotating pin [38]. Previous research [19] has identified four different zones for the produced FSSWed joints: SZ, the thermomechanically affected zone (TMAZ), the heat-affected zone (HAZ), and base material (BM). These four regions are typical characteristics of the FSSW cross-sectional specimens.

### 3.3. Grain Structure

**Figure 7:**
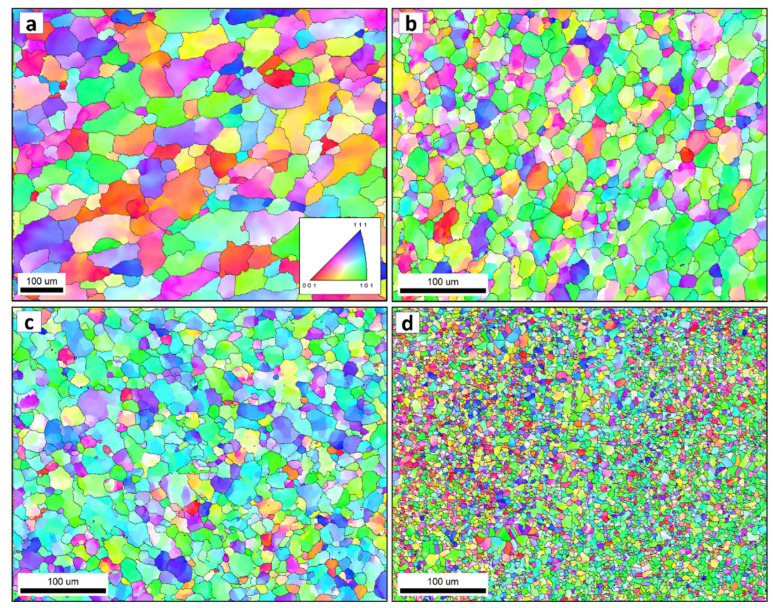
The IPF maps for the BM in (**a**) and the SZ of the FSSWed AA5052-H32 at a constant dwell time of 2 s and different tool rotation speeds of (**b**) 1500, (**c**) 1000 and (**d**) 500 rpm with the high angle grain boundaries greater than 15^o^ of misorientation superimposed in black lines.

**Figure 8:**
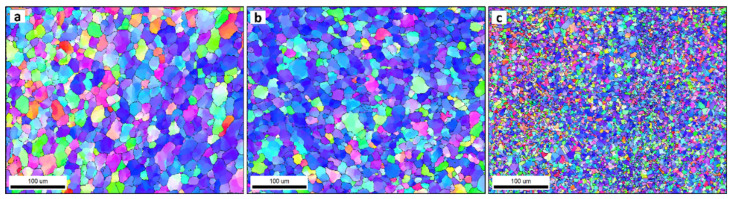
IPF maps after rotation to align the shear reference frame with the FSSW reference frame for the data presented in Figure 7 for different tool rotation speeds of (**a**) (1500 rpm), (**b**) (1000 rpm), and (**c**) (500 rpm).

**Figure 9:**
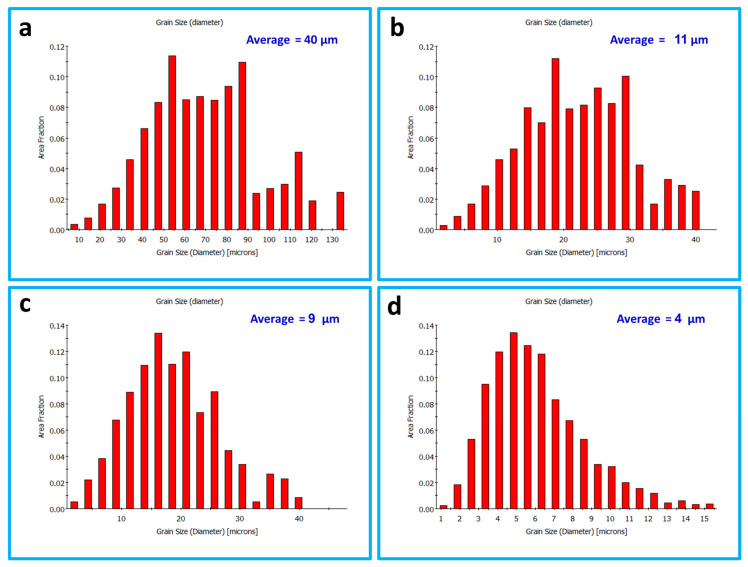
Grain size distributions obtained from the EBSD data presented in Figure 7 for the BM in (**a**) and the SZ of the FSSWed AA5052-H32 produced at a constant dwell time of 2 s and different tool rotation speeds of (**b**) 1500, (**c**) 1000, and (**d**) 500 rpm.

**Figure 10:**
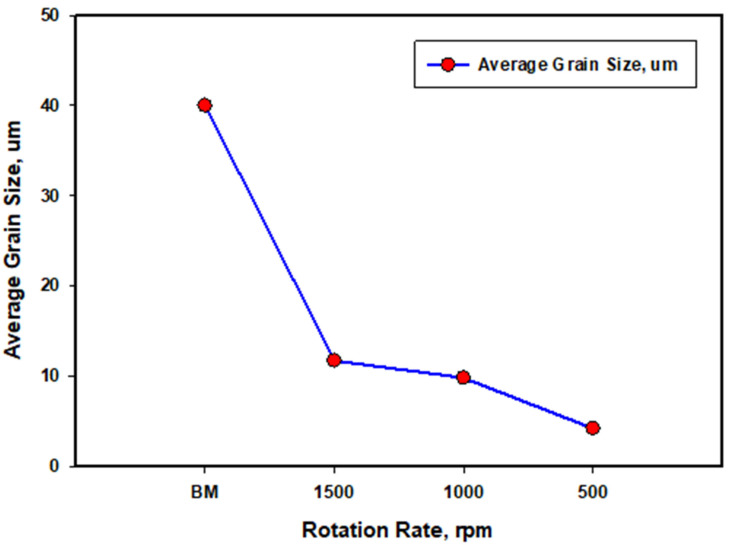
Average grain size variation in the BM to the different rotation speeds FSSWed joints.

Figure 7 shows the inverse pole figure coloring (IPF) maps for the BM in (a) and the SZ of the FSSWed AA5052 at a constant dwell time of 2 s and different tool rotation rates of (b) 1500, (c) 1000 and (d) 500 rpm with the high angle boundaries greater than 15° of misorientation in superimposed black lines. The IPF map presented in Figure 7a for the BM indicates the coarse grain structure with mixed grain orientation. After FSSW, this coarse grain structure is dynamically recrystallized into an equiaxed grain structure with smaller grain sizes caused by the high strain and high temperature experienced due to the severe friction between the tool and the material. It can be observed that the microstructure becomes finer when reducing the tool rotation rate from 1500 rpm (Figure 7b) to 1000 rpm (Figure 7c) and further to 500 rpm (Figure 7d). In terms of orientation, the maps are dominated by the <101> green orientation. It should be mentioned here that these are the raw EBSD data without rotation. It is known that the deformation reference frame during FSW/FSSW is not fixed and moves in a radial path with the tool rotation, which requires rotation of the EBSD data to align the deformation reference frame with the shear reference frame [60]. Figure 8a–c shows the IFP maps after applying the required data rotation, mainly around the normal direction (ND). Now, it can be observed that the maps are dominated mainly by the <111> blue orientation, which implies the alignment of the <111> direction with the normal direction. The grain size distribution histograms for the BM and the FSSWed materials are presented in Figure 9. The BM grain size ranges from about 5 µm up to 130 µm with an average of about 40 µm. Significant grain size reduction can be noted after FSSW, where the grain size in the joint produced at 1500 rpm ranges from about 1 µm to 40 µm with an average of 11 µm that has been reduced to an average of 9 µm after decreasing the tool rotation to 1000 rpm. However, more notable grain size reduction can be noted after reducing the tool rotation to 500 rpm, where the grain size ranges from 1 µm to 15 µm with an average of about 4 µm. Figure 10 shows the average grain size against the tool rotation rate. This grain refinement caused by reducing the FSSW tool rotation rate is mainly due to the reduction in the heat generated during the process. It was found (Section 3.2) that the reduction in the FSSW tool rotation from 1500 rpm to 500 rpm reduced the heat input from 4500 to 1525 J. This reduction in the heat input allows the exposure of the weld zone to a lower thermal cycle profile during and after FSSW [61].

**Figure 11:**
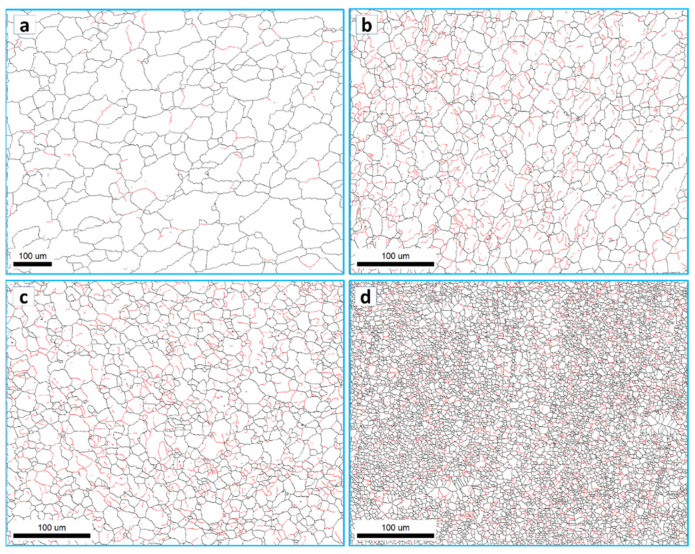
Grain boundary maps with HABs > 15° in black lines and low-angle boundaries 5° < LABs < 15° in red lines for the BM in (**a**) and the SZ in the FSSWed AA5052-H32 at a constant dwell time of 2 s and different tool rotation speeds of (**b**) 1500, (**c**) 1000, and (**d**) 500 rpm.

**Figure 12:**
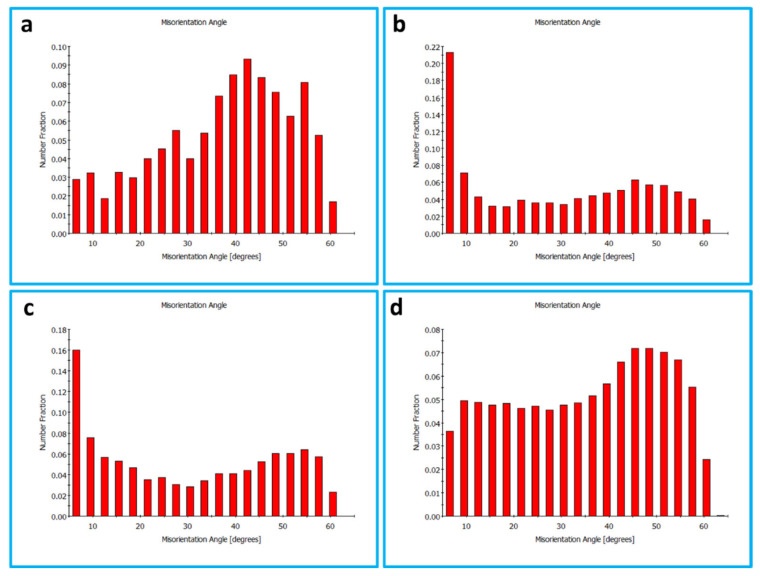
Misorientation angle distributions obtained from the EBSD data presented in Figure 7 for the BM in (**a**) and the SZ in the FSSWed AA5052-H32 at a constant dwell time of 2 s and different tool rotation speeds of (**b**) 1500, (**c**) 1000, and (**d**) 500 rpm.

The grain boundary maps are presented in Figure 11 for the BM (a) and the FSSWed joints produced at different tool rotation speeds of 1500 (b), 1000 (c), and 500 (d) with the high-angle boundaries (HABs) >15° shown in black lines and 15° > low angle boundaries (LABs) > 5° shown in red lines. HABs mainly dominate the BM GB map with only a slight fraction of LABs, whereas the GB maps of the FSSWed material consist of HAB and a high fraction of LABs. This microstructural feature indicates that the severe plastic deformation at high temperatures is associated with continuous dynamic recrystallization, forming a new grain structure [62]. The cooling afterward preserves a high fraction of substructures. The misorientation angle histograms presented in Figure 12a–d compare the BM and FSSWed materials, which clearly show the dominance of the HABs in the BM and the high fraction LABs in the FSSWed material, especially in the histograms for the joints produced at 1500 and 1000 rpm. In contrast, the joint produced at 500 rpm dominates the HABs again due to the extremely fine grain structure formed.

### 3.4. Crystallographic Texture

**Figure 13:**
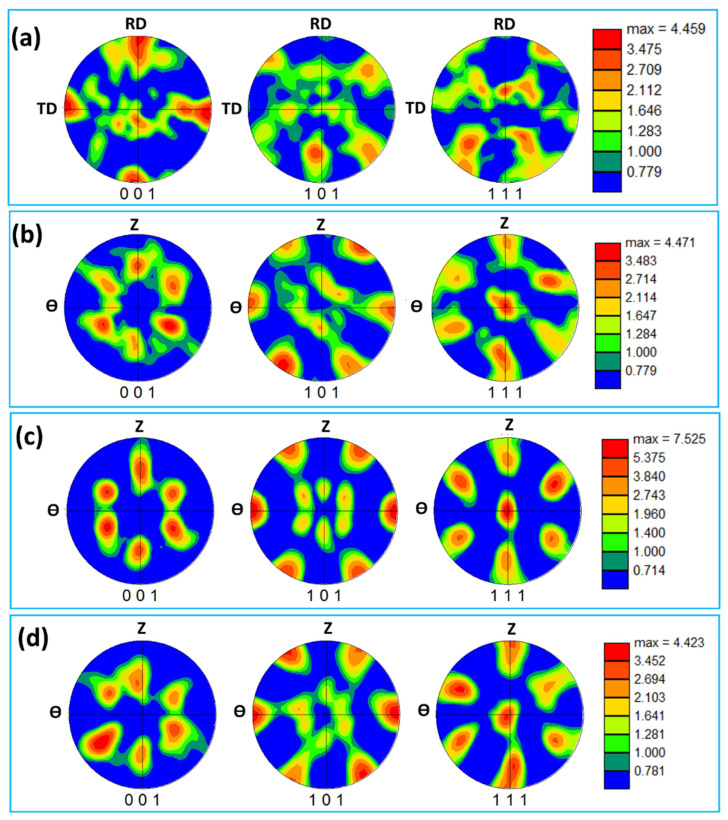
The 001, 101, and 111 pole figures calculated using the EBSD data presented in Figure 7 for the BM in (**a**) and the SZ in FSSWedAA5052-H32 at a constant dwell time of 2 s and different tool rotation speeds of (**b**) 1500, (**c**) 1000, and (**d**) 500 rpm.

**Figure 14:**
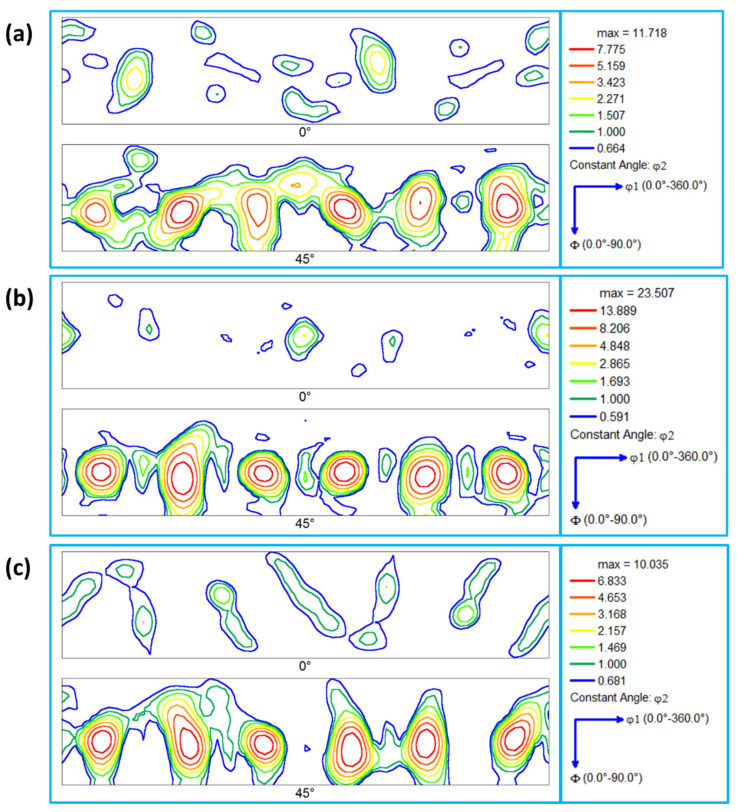
Orientation distribution function (ODF) sections at φ2 = 45° and φ2 = 0° calculated using the EBSD data presented in Figure 7 for the BM in (**a**) and the SZ in the FSWed AA5052-H32 at a constant dwell time of 2s and different tool rotation rates of (**a**) 1500, (**b**) 1000, and (**c**) 500 rpm.

**Figure 15:**
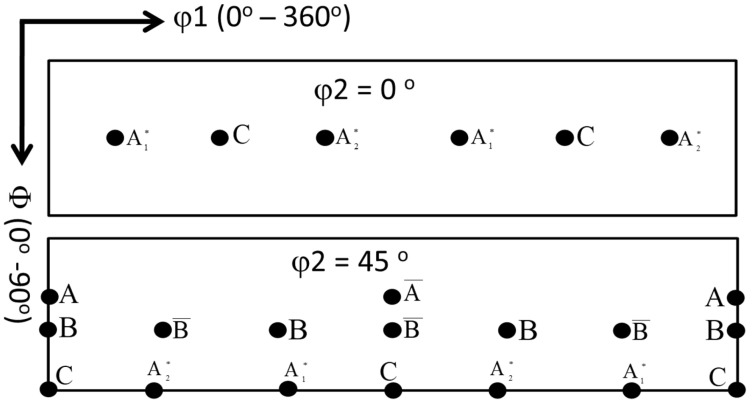
Schematic showing the ODF sections at φ2 = 45° and φ2 = 0° with the simple shear ideal texture components presented at their ideal positions.

The deformation of aluminum during FSW/FSSW is mainly dominated by simple shear deformation that mainly results in a simple shear texture due to the alignment of the {111} slip plane with the shear plane and/or alignment of the <110> slip direction with the shear direction [63]. Table 1 shows the different types of texture fibers that can develop upon shear deformation depending on the extent of strain experienced. It can be observed that the rotated cube texture of the BM (Figure 13a) has completely changed after FSSW in the NG zone into the simple shear texture with the B/-B texture components existing at their ideal position in the pole figures, as shown in Figure 13b–d. It can be observed that the texture intensity for the joint produced at 1500 rpm (Figure 13b) is about 5 times random, that it increased to about 8 times random for the joint produced at 1000 rpm (Figure 13c), and that the texture intensity reduced again to about 4 times random for the joint produced at 500rpm. At the high rotation rate, the heat input is maximum and the temperature at the weld zone is expected to be high, which can accelerate the restoration mechanisms, mainly recovery, and recrystallization. It should be mentioned here that the obtained EBSD data was rotated to align the deformation reference frame (TD: transverse direction, WD: welding direction, and ND: normal direction) with the shear reference frame (θ, z, and r). The rotations are mainly about the ND and TD. The reason for these rotations has been reported by previous studies on the texture analysis of friction stir welded aluminum [60,64]. Fonda and Bingert [64] rotated the pole figures 7° about the WD, 7° about the TD, and 10° about the ND to align them with the ideal shear deformation reference frame. Ahmed et al. [60] reported a systematic rotation in the texture across the whole SZ in their investigation of texture across the SZ in friction stir welded AA6082 of 30 mm thickness. Thus, two rotations are required to analyze the texture components relative to the shear reference frame across the whole SZ: rotation about the ND to take into account the shear reference frame rotation about the ND and rotation about the TD to take into account the shear reference frame tilt due to the tapering of the probe [60]. In this work, the pole figures have been rotated about the ND and TD to obtain the texture components at their ideal positions. The orientation distribution function (ODF) was used to create sections at φ2 = 45° and φ2 = 0° for the FSSWed samples at different rotation rates of 1500 rpm (Figure 14a), 1000 rpm (Figure 14b), and 500 rpm (Figure 14c). From these ODF sections, the dominance of the B/-B texture components can be observed. In addition, the joint produced showed the highest texture intensity. However, the B/-B texture components are still slightly shifted about 22.5° from their ideal position when compared to the ideal texture components positions presented schematically in Figure 15 for ODF sections at φ2 = 45° and φ2 = 0°. Also, Table 2 gives the partial fibers and ideal components of simple shear texture in fcc metal. 

### 3.5. Hardness Evaluation

**Figure 16:**
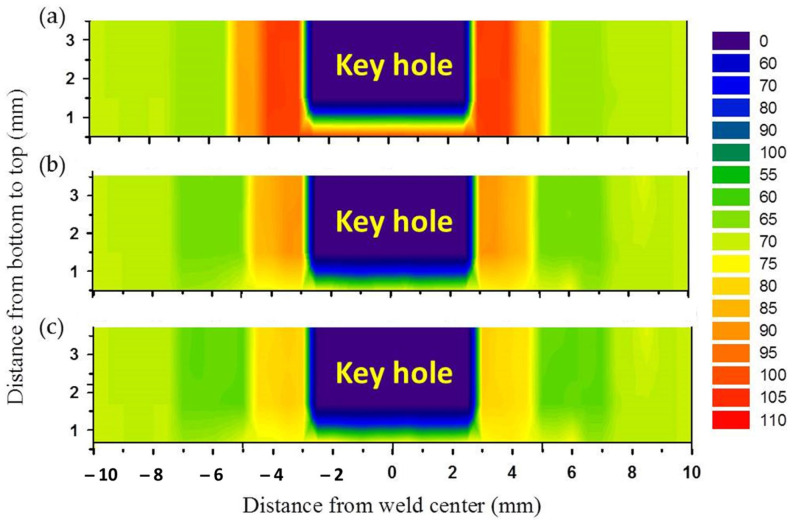
Hardness contour maps for the AA5052-H32 FSSWed specimens processed at rotation speeds of (**a**) 500, (**b**) 1000, and (**c**) 1500 rpm and a constant dwell time of 2 s.

**Figure 17:**
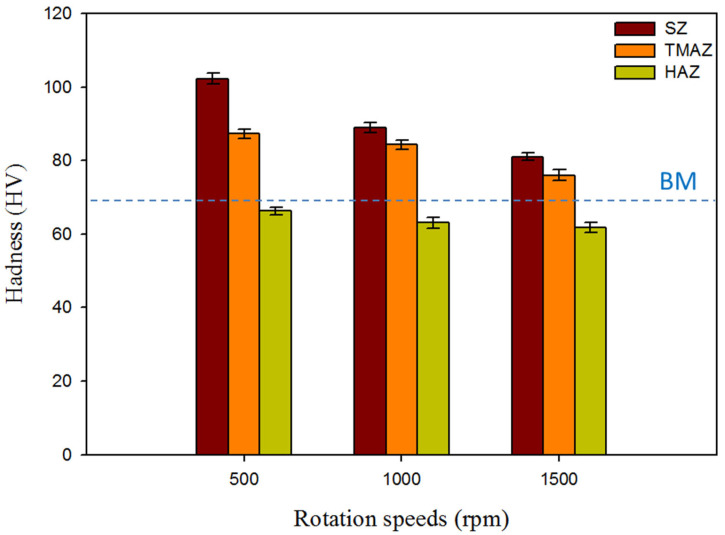
Average hardness values in the welded zones (SZ, TMAZ, and HAZ) as a function of the applied various rotational speeds and a constant dwell time of 2 s.

The hardness of the produced AA5052-H32 FSSW lap joints was evaluated using transverse cross-sections. The measurements were displayed on a contour map with different colors representing varying hardness levels. Figure 16 shows the hardness contour maps of the FSSWed specimens processed at a 2 s dwell time and various tool rotation speeds of 500, 1000, and 1500 rpm. The cylindrical pin achieved a symmetrical hardness distribution with regard to the center line of the FSSW keyhole for all the applied tool rotation speeds of 500, 1000, and 1500 rpm, as given in Figure 16a–c, respectively. Furthermore, the hardness values of the welded regions, SZ, and TMAZ in the AA5052-H32 FSSWed joints were significantly higher at all the used rotation speeds than the BM. However, depending on the thermal exposure cycle (total heat input), the hardness of the HAZ is somewhat less than that of the AA5052-H32 BM, as shown in Figure 16 and Figure 17. These results agree with those previously reported by other researchers [27,29]. Tiwan et al. [27] observed that the SZ in the FSSWed AA5052-H112 Al alloy joints had higher hardness than the TMAZ and HAZ. They attributed this improvement in hardness to the grain refining process owing to dynamic recrystallization. Dynamic recrystallization occurs when crystalline materials are plastically deformed at high temperatures [66]. Likewise, Kwon et al. [66] ascribed an enhanced hardness of the SZ in the FSSW A5052-O joints over its BM to grain refining. In the current work, the minimal hardness values found in the HAZ for all the AA5052-H32 FSSW joints can be related to the grain structure and strain hardening release. However, the increased hardness of the SZ was mostly due to the development of dynamically recrystallized equiaxed fine grains and the potential intermetallic fragmentation process that occurred during the stirring action [29]. As seen in Figure 17, the TMAZ has a lower hardness than the SZ but a higher hardness than the HAZ. It can be concluded that the high dislocation density generated by extreme plastic deformation during the welding process promotes the increased hardness of the TMAZ above the HAZ. At a constant dwell time of 2 s, the FSSW joints fabricated at 500 rpm show the highest average hardness values of 102.3 ± 3 in the SZ compared to the BM (69 ± 2) and the other joints produced at 1000 rpm (89.2 ± 2) and 1500 rpm (81.3 ± 2), as shown in Figure 17. This decrease in hardness in the SZ with increasing rotation speed is ascribed to the raised introduced heat input.

### 3.6. Tensile Test Results and Evaluation of Fracture Surfaces

**Figure 18:**
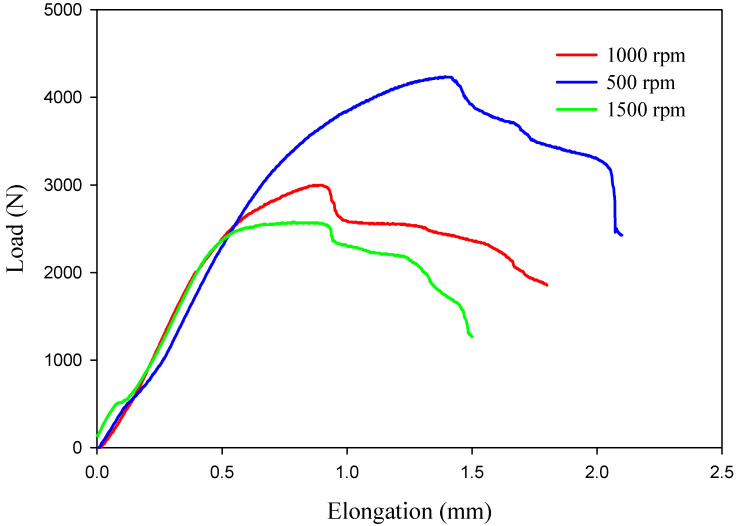
Tensile–shear curves in terms of load against the elongation of the AA5052-H32 welds produced at different rotation speeds (500–1500) and a 2 s dwell time.

**Figure 19:**
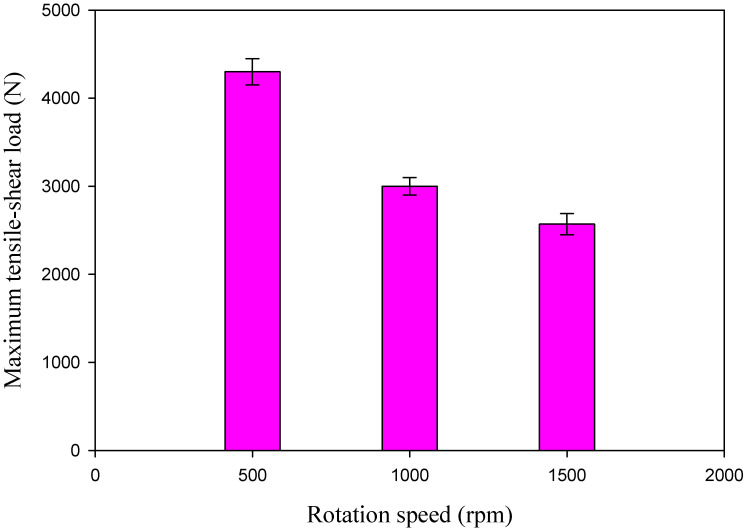
Maximum tensile–shear load of the AA5052-H32 FSSW joints versus the tool rotation speed.

When designing new types of automobiles, aircraft, and ships, engineers must consider the efficiency of spot-welded connections. Thus, the FSSWed AA5052-H32 joints were subjected to a tensile shear test to determine the maximum load-carrying capacity of each joint and to optimize the processing parameters. It was found that the FSSW process parameters influence the maximum tensile shear value of the spot-welded connection [67,68]. Figure 18 shows load elongation curves for the tensile–shear test results of the spot-welded specimens manufactured at different rotation speeds of 500, 1000, and 1500 rpm and a 2 s dwell time. Figure 19 depicts the maximum value of the tensile–shear load of the AA50520-H32 FSSW specimens against the applied different rotation speeds. It can be noted that the FSSW processing parameters (2 s dwell time and 500 rpm) give the highest tensile–shear load of 4330 ± 120 N when compared to the tensile–shear loads of the processed joints at 1000 rpm (3000 ± 100 N) and 1500 rpm. (2569 ± 90 N), as shown in Figure 18 and Figure 19. This increase in performance in terms of the maximum load carrying capacity of the joint processed at 500 rpm may be attributed to the largest welded zone [35] and the highest hardness value of the SZ compared to the other joints. In addition, higher rotation speeds of 1000 and 1500 rpm (higher heat input energy) achieve sufficient mixing between the two welded aluminum strips, and the extra heat may cause softening in the welded area and increase the HAZ area, resulting in a lower joint strength than that processed at 500 rpm, as shown in Figure 10. Overall, it can be remarked that the tensile results confirmed the hardness results for all the produced joints.

**Figure 20:**
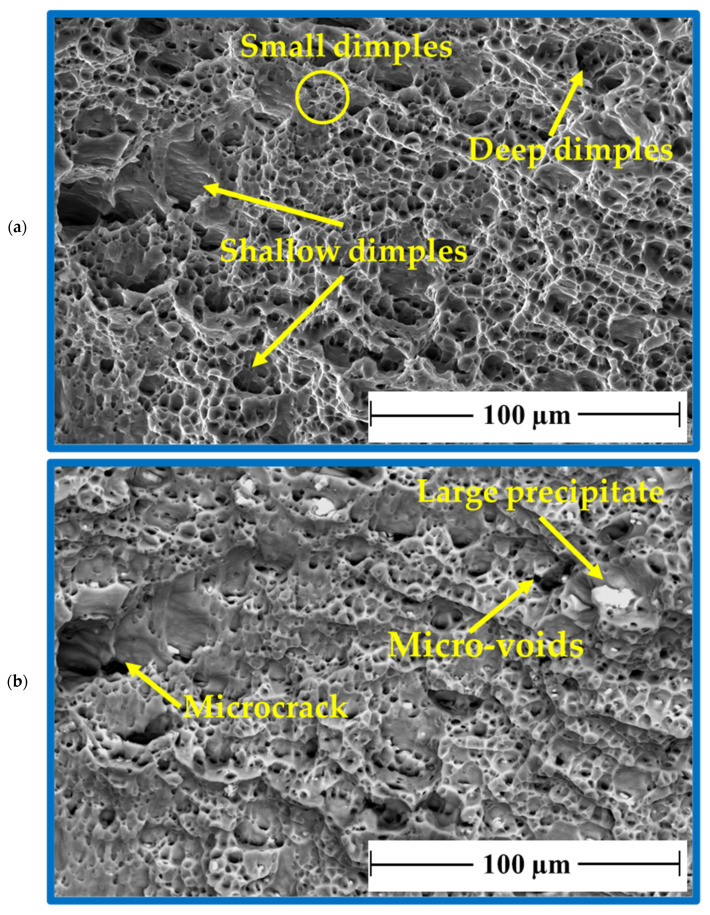
Fracture surface SEM images showing the AA5052-H32 BM (**a**) ETD and (**b**) VCD.

After tensile testing, the fracture surface of AA 5052-H32 BM was examined using two SEM detectors, i.e., the Everhart–Thornley Detector (ETD) and a low K-Volt High-Contract Detector (vCD), to study the fracture surface features, as shown in Figure 20a,b, respectively. There are two fracture modes: mainly ductile and partly brittle. The AA505-H32 aluminum alloy matrix showed a ductile fracture in different dimples with shapes and sizes (Figure 20a), whereas the brittle fracture mode was given by the large precipitates of Al_3_Fe and Mg_2_Si [69]. Furthermore, micro-cracks and micro-voids are also seen as a result of the precipitate’s pull-out mechanism from the fracture surface (Figure 20b).

**Figure 21:**
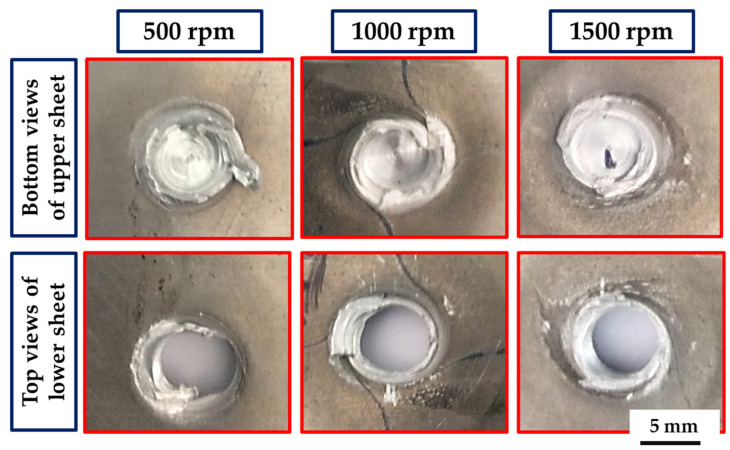
Photographs showing the fracture location of the AA5052-H32 FSSWed joints after tensile–shear testing.

**Figure 22:**
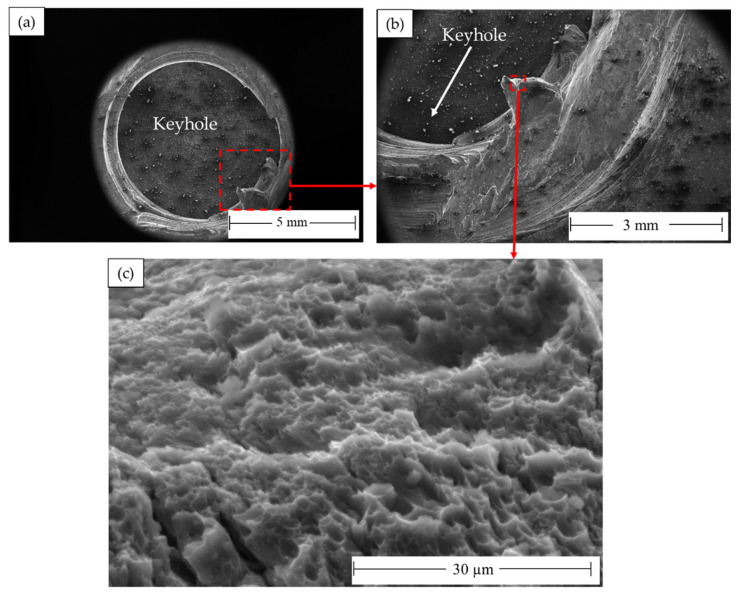
Fracture surface SEM images showing (**a**) the top view of the separated lower AA5052-H32 sheet FSSWed at 2 s and 500 rpm after failing during the tensile test and (**b**,**c**) different magnifications.

**Figure 23:**
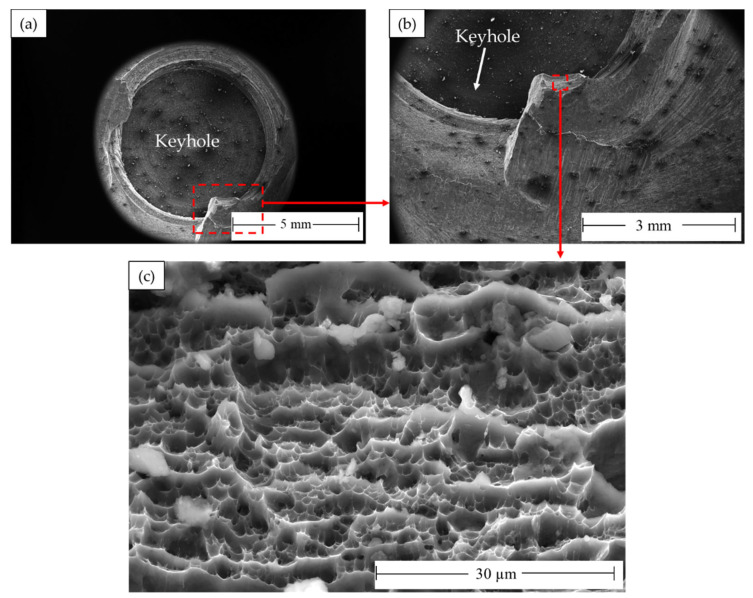
Fracture surface SEM images showing (**a**) the top view of the separated lower AA5052-H32 sheet FSSWed at 2 s and 1000 rpm after failing during the tensile test and (**b**,**c**) different magnifications.

**Figure 24:**
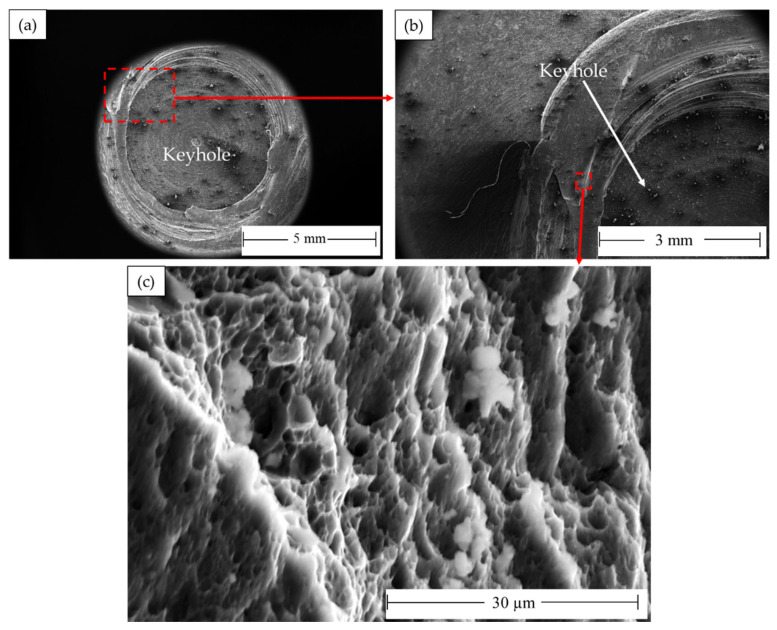
Fracture surface SEM images showing (**a**) the top view of the separated lower AA5052-H32 sheet FSSWed at 2 s and 1500 rpm after failing during the tensile test and (**b**,**c**) different magnifications.

The processed AA 5052-H32 lap joints are detached during the tensile test. Figure 21 shows photo images of the failed joints after tensile testing. As can be observed, all the produced joints start to fracture at the edge of the SZ and develop along the depression in the upper joint strip. After the joint was separated into two parts, the upper sheet in the SZ was left in the lower sheet to form a button shape. Figure 22, Figure 23 and Figure 24 show fracture surface SEM images of the separated lower sheets after the tensile–shear test for the 5052-H32 FSSW specimens processed at different rotation speeds of 500 rpm (Figure 22a–c), 1000 rpm (Figure 23a–c), and 1500 rpm (Figure 24a–c) and a 2 s dwell time. The tensile–shear mechanism caused the failure in the three FSSW joints. Microcracks start at the partly bonded area at the tip of the hook and preferentially propagate horizontally at the weld joint interface during tensile–shear testing, shearing the SZ and leading to failure. Compared to the AA5052-H32 BM, the fracture surfaces on the three welds at the lower sheets show typical ductile characteristics in the form of small equiaxed dimples, indicating grain refinement due to dynamic recrystallization through the SZ during the welding process.

## 4. Conclusions

In this work, 2 mm thick AA5052-H32 strips were FSSWed at various tool rotation speeds of 1500, 1000, and 500 rpm while the dwell time was fixed at a constant value of 2 s. The macro- and microstructure features, crystallographic texture, and mechanical properties of produced welds were assessed, and the following conclusions were drawn:

- In comparison to the AA5052-H32 BM, all the FSSWed AA5052-H32 lap joints showed an improvement in the hardness of the SZ and TMAZ. Moreover, the SZ displayed higher values of hardness than those observed for the TMAZ and HAZ. In addition, the hardness of the weld zone decreases with increasing rotation speed from 500 to 1000 rpm.
- Compared to the AA5052-H32 BM grain size (40 µm), there was significant grain refining in the SZ of the produced FSSW joints. For the FSSWed joints processed at 1500, 1000, and 500 rpm, the grain size values in the stir zones were 11, 9, and 4 µm, respectively. The grain size decreased with decreasing heat input.
- The texture is mainly dominated by B/-B simple shear texture components with little effect of the rotation rate either on the texture components or the intensity of the texture.
- The spot-welded joint produced at 500 rpm and 2 s showed the highest SZ hardness of 102.3 ± 3 HV0.5 and tensile–shear load value of 4330 ± 30 N among the produced joints.
- When compared to the AA5052-H32 BM, the fracture surfaces on the three welds at the lower sheets showed typical ductile characteristics in the form of small equiaxed dimples, indicating grain refinement due to dynamic recrystallization through the SZ during the FSSW process.
- Finally, this work opens a fruitful field for researchers to work towards manufacturing spot welding joints at a low tool rotation speed equal to or less than 500 rpm and a short time of 2 s for different aluminum alloys, which represents a significant industrial goal to reduce energy use and production time.

## Figures and Tables

**Table 1:** The chemical composition the AA5052-H32 Al alloy.

Elements	Zn	Fe	Ti	Si	Mg	Cr	Mn	Cu	V	Al
(in wt. %)	0.200	0.258	0.018	0.127	2.490	0.195	0.090	0.001	0.001	Bal.

**Table 2:** Partial fibers and ideal components of simple shear texture in face-centered cubic (FCC) metals [63,65].

A/B Fiber	Shear Plane	Shear Direction	Euler Angles (°)		
	(hkl)	<uvw>	φ_1_	Φ	φ_2_
$A_{1}^{*}$	$\left\{11\bar{1}\right\}$	$\langle 2\bar{1}1\rangle$	35.26/215.26	45	0/90
			125.26	90	45
$A_{2}^{*}$	$\left\{11\bar{1}\right\}$	$\langle 112\rangle$	144.74	45	0/90
			54.74/234.74	90	45
$\bar{A}$	$\left\{11\bar{1}\right\}$	$\langle 1\bar{1}0\rangle$	0	35.26	45
$\bar{A}$	$\left\{11\bar{1}\right\}$	$\langle 101\rangle$	180	35.26	45
**B**	{112}	$\langle 1\bar{1}0\rangle$	0/120/240	54.74	45
$\bar{B}$	$\left\{\bar{1}\bar{1}\bar{2}\right\}$	$\langle 1\bar{1}0\rangle$	60/180	54.74	45
**C**	{001}	$\langle 1\bar{1}0\rangle$	90/270	45	0/90
			0/180	90	45

## Data Availability

Data will be available upon request to the corresponding author.
